# Dielectric permittivity extraction of MoS_2_ nanoribbons using THz nanoscopy

**DOI:** 10.1515/nanoph-2025-0060

**Published:** 2025-04-25

**Authors:** Henrik B. Lassen, William V. Carstensen, Denys I. Miakota, Ganesh Ghimire, Stela Canulescu, Peter U. Jepsen, Edmund J. R. Kelleher

**Affiliations:** Department of Electrical and Photonics Engineering, 5205 Technical University of Denmark, DK-2800 Kongens Lyngby, Denmark

**Keywords:** near-field, s-SNOM, THz spectroscopy, nanomaterials, MoS_2_

## Abstract

The nanoscale optical properties of high-quality MoS_2_ nanoribbons are investigated using THz nanoscopy based on a scattering-type scanning probe. The nanoribbons comprise a multilayer core, surrounded by monolayer edges. A featureless complex permittivity spectrum covering the range 0.6–1.6 THz is extracted from experimental time-domain measurements through a minimization procedure, adopting an extended finite-dipole model of the probe–sample interaction. Real-space mapping of the nanoribbon reveals variations in the local permittivity down to the instrument-limited resolution, on the order of 30 nm. Clustering analysis statistically identifies regions of lower apparent permittivity that we attribute to a high curvature at the edges of the nanoribbon causing an increase in local material strain or cross-talk in the measured signal with topography-induced measurement artifacts. The core of the nanoribbon contains two regions that follow tightly distributed, but slightly shifted Gaussian statistics in complex permittivity space, with the real part mean of both distributions lying around 5.4 and compatible with literature values of the static permittivity of thin-film MoS_2_ reported previously. Our results show that the nanoribbons exhibit a modest degree of dielectric variation at the nanoscale that could be explained by heterogeneous doping or variations in the local defect density. We believe that our approach could be useful for the direct real-space measurement of dielectric disorder in other low-dimensional semiconducting material systems.

## 1 Introduction

The dielectric permittivity of a material is a measure of its response to an electric field and is a frequency-dependent complex quantity. It is, therefore, a fundamental property of the material, governed by a specific chemical and structural composition, required in the design of semiconductor electronics. Recently, dielectric disorder in nanoscale systems – fluctuations in local complex permittivity on optical length scales – has been identified to contribute strongly to variations in the optoelectronic performance and transport properties of two-dimensional (2D) materials [1]. Transition-metal dichalcogenides (TMDs) are a family of 2D layered crystals, a subset of which are semiconducting and well-known to possess distinct properties in their mono- and few-layer form, namely a transition from indirect to direct electronic bandgap, compared to bulk. This behavior marks out several TMDs in particular, including MoS_2_, WSe_2_, MoSe_2_, and more, as attractive materials for photonic and optoelectronic device applications, such as light-emitting diodes, photodetectors [2], and photovoltaics [3], among others, due to an increased efficiency of light–matter interaction driving a stronger photoluminescence and greater light absorption. A further lowering of the physical dimensions can dramatically influence the intrinsic material properties [4], [5], [6], boosting photoresponsivity and nonlinear effects [7], [8]. The potential of TMD-based nanostructures, including MoS_2_ nanoribbons, has pushed the development of numerous TMD synthesis methods forward and emphasized the importance of dedicated characterization of TMD properties [9], [10], [11], [12], [13].

Angle-dependent Raman [14], [15] and photoluminescence spectroscopies [16], [17] have been widely applied in the study of layered materials, providing rich information on crystallographic structure including anisotropy, vibrational modes, strain, band structure, and defects; neither technique, however, directly captures the dielectric function and both are typically restricted in their ability to resolve features in materials or heterogeneities in optical properties smaller than is allowed by the diffraction-limited focusing of visible light (typically several hundreds of nanometers at best). Ellipsometry [18] and impedance spectroscopy [19], on the other hand, can directly record the frequency-dependent dielectric permittivity (albeit in very distinct frequency regions, either in the visible/near-infrared or radio frequency range), but are again subject to either the diffraction limit of optical systems or determine only an average macroscopic material response. Similarly, time-domain methods in the infrared and low-frequency terahertz (THz) region of the electromagnetic spectrum, notably THz time-domain spectroscopy (TDS) [20], have proven effective for material characterization because THz radiation, in particular, is commensurate with the energy scale of free carriers in materials leading to a strong interaction. THz-TDS has been widely applied to semiconducting and metallic thin films, including graphene, to investigate complex permittivity and conductivity, without contacts [21], [22]. However, the challenge is again the spatial resolution, which is now limited to several tens or even hundreds of micrometers because of the low frequency (typically 0.3–3 THz) of the probing radiation.

Scanning probe microscopy (SPM) techniques, such as atomic force microscopy (AFM) using sharpened (conductive) tips with an apex radius on the order of several tens of nanometers, can be used to explore material properties with significantly enhanced spatial resolution and are, therefore, suitable for studying nanostructured and heterogeneous materials locally. Electrostatic force microscopy (EFM) – an AFM-based technique exploiting the change in capacitance between a voltage-biased probe and the sample surface to infer dielectric properties – is one example that has been applied to thin-film TMDs for recovery of the local electrostatic dielectric constant at (or very close to) DC [23], [24] but importantly does not provide information of dielectric relaxation behavior in even a limited frequency range. Scattering-type scanning near-field optical microscopy (s-SNOM) [25], [26], essentially a modified AFM with an external light source, equipped with infrared or THz illumination has become a powerful technique for nanoscale imaging and spectroscopy in a technologically relevant frequency range, combining the benefits of both sensitivity to free-carrier absorption of far-field THz-TDS, with the nanoscale resolution of other surface probes. The spatial resolution of s-SNOM is essentially agnostic to the wavelength of the illuminating light source [27] and is rather defined by the radius of the scanning probe (or tip) at its apex. THz-SNOM, therefore, probes a similar volume in real-space compared to SNOM illuminated with visible light. Significantly, however, the mismatch between the free-space wave-vector and the (in-plane) momenta of tip-scattered light at THz frequencies can reach 10^3^, meaning THz-SNOM probes deeply into the near-field regime. When the light source is a broadband THz pulse covering a wide frequency range, THz-enabled SNOM becomes a potent tool for nanoscopy of advanced materials. The challenge of SPM methods is often to accurately represent the tip–sample interaction. Quantitative extraction of material properties from experimental near-field scattering data in s-SNOM is often a formidable task, but several attempts in the recent literature have successfully applied an underlying model of the physical tip–sample system that can be inverted to relate fundamental parameters to measurable quantities [28], [29], [30], [31], [32], without needing to make model assumptions about the dielectric function, and in the case of nanoscopy its spectral dependence. The inversion can be performed with an analytic approximation [29] or using an iterative numerical minimization algorithm [33], with both demonstrated to yield robust and reliable output. Here, we use ultrafast THz pulses with a useful bandwidth spanning 0.6–1.6 THz for nanoscopy in an s-SNOM setup to interrogate MoS_2_ nanoribbons and recover their complex dielectric response using a numerical minimization procedure based on an extended finite-dipole model for a layered material system [34], [35], [36]. Subsequent nanoscale THz imaging of the nanoribbon and a clustering analysis of the spatially dependent dielectric data is used to identify regions strongly influenced by edge effects where high surface curvature could indicate an impact from local strain or cross-talk from topographic artifacts, together with regions in the core of the nanoribbon where we observe two clearly distinguishable areas defined by tightly distributed Gaussian statistics that we believe capture directly nanoscale variations in the materials dielectric response.

## 2 Materials and methods

### 2.1 Nanoribbon growth

**Figure 1:**
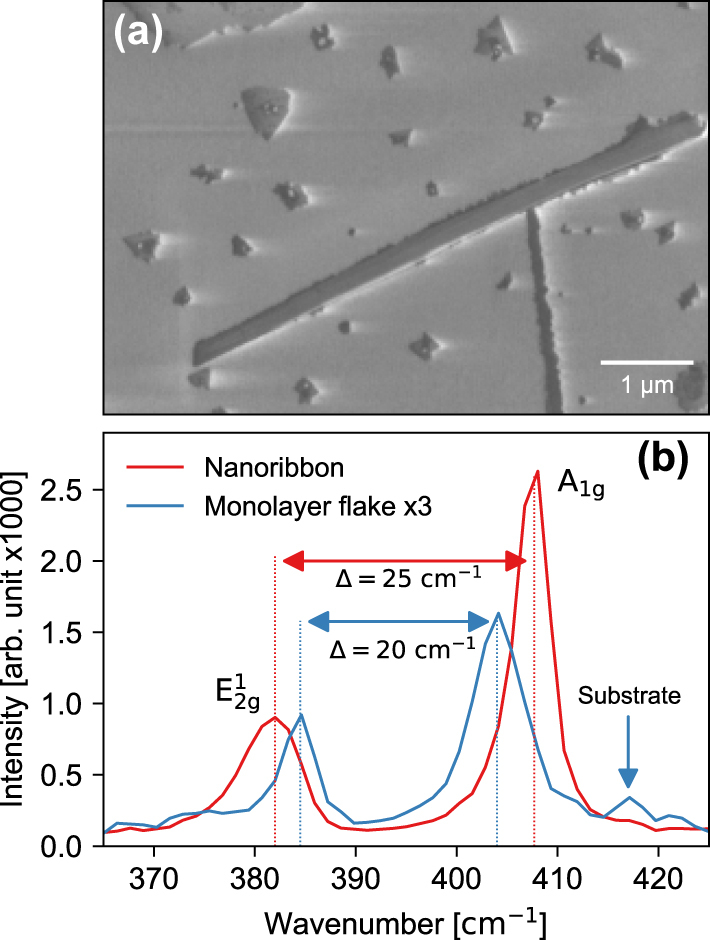
Characteristic nanoribbon properties. (a) SEM image of a characteristic MoS_2_ nanoribbon surrounded by triangular 2D and 3D crystallites of MoS_2_ and (b) Raman spectrum obtained from a region of the MoS_2_ nanoribbon (blue) and on a 2D MoS_2_ crystal (orange). The signal from monolayer MoS_2_ has been multiplied by a factor of 3 for better representation.

The MoS_2_ nanoribbons were grown on a *c*-cut sapphire substrate. Synthesis was a two-step process in which ultra-thin oxide films of MoO_3_-x (with many oxygen vacancies) grown by pulsed laser deposition were sulfurized at the second step in the presence of a NaF layer, the details of the process can be found in our previous works [8], [37]. The technique is similar to that described by Li et al. in Ref. [5] and shares many similar ideas on liquid phase creation and vapor–liquid–solid phase reaction. Briefly, the growth process evolves via the formation of the Na–Mo–O liquid phase, which mediates the formation of MoS_2_ multilayer nanoribbons in a sulfur-rich environment [5], [8], [37]. The nanoribbons crystallize predominantly in the 2H stacking orientation [8]. Due to strong in-plane covalent bonding and weak out-of-plane van der Waals (vdW) interactions, layered 2D materials possess a strongly anisotropic dielectric tensor [38], with distinct in-plane and out-of-plane components. Figure 1a shows a secondary-electron scanning electron microscope (SEM) image of a typical MoS_2_ nanoribbon on sapphire with a length of 10 μm and a width of less than 0.5 μm, resulting in a length-to-width ratio of nearly 20. Isolated 2D and 3D crystallites of MoS_2_ can also been seen in the SEM image. A typical Raman spectrum of such samples taken using a laser with a wavelength of 532 nm is shown in Figure 1b. The spectrum shows two major characteristic Raman peaks of MoS_2_ arising from the in-plane $E_{2g}^{1}$ and out-of-plane *A*_1*g*_ Raman modes. The peak position difference between the Raman modes of 25 cm^−1^ usually denotes a bulk MoS_2_ response, which is in a good agreement with previous reports [39]. It should be noted that a strong $E_{2g}^{1}$ Raman peak shift can be experienced for the as-grown samples due to the strain presence in as-grown MoS_2_ nanoribbons. Here, we exclude the presence of residual strain in the MoS_2_ nanoribbon, because for the MoS_2_ nanoribbons grown, the strain can be released via rupture of the nanoribbons or the folds of the constituent MoS_2_ nanoribbon top layers [37], which can be seen in Figure 1a, further supported by analysis of the AFM topography map discussed later (see Supplementary Information), where the core of the nanoribbon shows a very small surface curvature that has been associated with low local strain [40].

**Figure 2:**
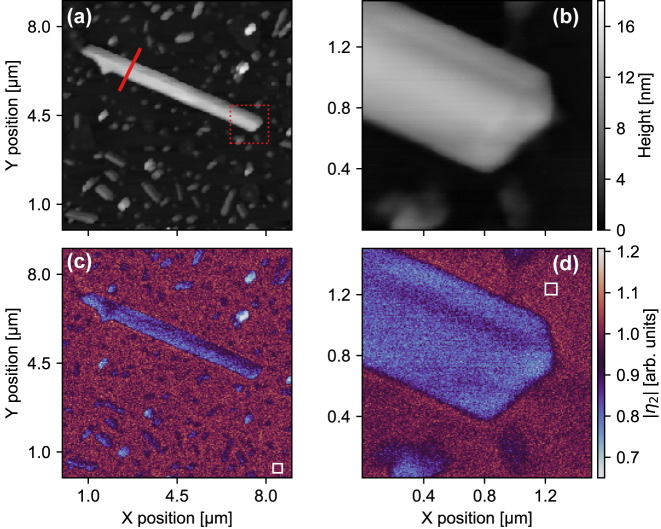
Topography and scattering contrast maps. (a) AFM images of the selected MoS_2_ nanoribbon and (b) a zoom-in scan of one end of the nanoribbon as indicated by the red dotted square in (a). The red line in (a) indicates the position of the spectroscopic line scan (THz nanoscopy). The corresponding white-light mode imaging maps, (c) overview and (d) zoom-in, of the near-field contrast (second-order demodulated scattered signal). The white squares in (c) and (d) indicate the areas used to generate a mean substrate response for normalization.

The exact dimensions of the MoS_2_ nanoribbon used in this study can be inferred from the AFM images in Figure 2a and b, showing an overview and zoom-in (corresponding to the area indicated by the dotted red box in Figure 2a). The thickness of the nanoribbon (as confirmed by our AFM measurements, *e.g.*, Figure 2a) varies between approximately 10 nm and 15 nm, correspondingly the multilayer core of the nanoribbon consists of >15 layers suggesting the properties approach that of the bulk crystal. Notably, monolayer edges of the MoS_2_ nanoribbon that can be observed in the SEM imaging are not visible with AFM. It should be noted that the SEM and AFM were not performed on the same nanoribbon. The reason for discrepancies between the SEM and AFM images can be twofold: (i) these edges are not pronounced in the selected nanostructure or (ii) a limitation of the instrument: compared to SEM, AFM has a poor depth of field, limiting its ability to resolve features with large height differences. The abrupt change in thickness between the multilayer nanoribbon (15L) and the adjacent monolayer (1L) could make the monolayer edge undetectable by AFM.

### 2.2 Near-field imaging and nanoscopy

The near-field microscope used is a commercial instrument (Attocube THz-NeaSCOPE) equipped with an integrated THz time-domain spectroscopy module (Attocube/Menlo Systems TeraSmart). THz pulses with a useful bandwidth covering the spectral range 0.6–1.6 THz are generated and coherently detected by a photoconductive antenna pair. A conductive AFM tip (with a shank length of 80 μm and typical average tip radius of <40 nm, Rocky Mountain Nanotechnology, 25PtIr200B-H), operated in noncontact tapping mode (at a nominal frequency of 80 kHz), acts as a nanofocusing surface probe, allowing for simultaneous capture of sample topography together with measurements of the scattered near-field signal in a single scan of the sample. Measurements are performed in a nitrogen-rich environment to minimize the presence of water-vapor absorption lines in the detected THz spectra. Background removal is accomplished by demodulation of the scattered field to recover the near-field signal that is most pure in higher overtones of the tapping frequency (typically orders *m* = 2–4 are used for data retrieval due to diminishing signal-to-noise ratio affecting data quality at orders above 4) [25]. The scattered THz waveform is detected in the time domain in two distinct modes of detection that are common to other THz-SNOM systems. Firstly, white-light (WL) detection offers rapid imaging of the surface by resolving the electric field at the principal peak of the waveform (this corresponds to a spectrally integrated near-field response [41]). In this case, the WL signal, $\hat{E}$ , is associated with both the amplitude and phase of the scattered radiation. In contrast, TDS mode (or nanoscopy) records the full scattered THz waveform in time from which the amplitude and phase spectra can be recovered using Fourier transformation, as is widely established in far-field THz-TDS [42]. The incident electric field is *p*-polarized to maximize the scattered signal [43] and focused to the tip–sample interface using reflective optics at an angle of 30° to the surface normal. Due to the axisymmetry and elongated geometry of the AFM probe, it is widely known that the tip is dominantly polarizable parallel to its long axis, resulting in a larger out-of-plane polarizability [44]. Consequently, although the scattered signal measured due to the tip–sample dipole is affected by the in-plane and out-of-plane components of the dielectric permittivity tensor [45], the large in-plane momentum of fields scattered by the probe makes the technique most sensitive to changes in the out-of-plane component. Thus, in the discussion of our results, we consider an effective dielectric permittivity, acknowledging that this can be a complex mix of diagonal tensor elements for a uniaxial anisotropic crystal. We note, however, that under certain conditions, the effective permittivity is separable: by exploiting parametrization and information from specific partially screened substrate resonances, the dielectric tensor of bulk 2H-WSe_2_ was determined in a specific frequency range in the mid-infrared [45]. Other efforts to recover dielectric tensor elements from s-SNOM data have included the use of guided modes in the near-infrared [46] or specially adapted probes [44].

### 2.3 Modeling and inversion of the scattering problem

The scattering of a low-frequency electric field by the combined tip–sample system can be described by several self-consistent, quasi-electrostatic models of the problem [25], [31], [35], together with extensions to allow for layered structures, including thin films on a bulk substrate [30], [34], [36], based on a transfer matrix formalism [47]. The finite-dipole model (FDM) [35], and its extensions for layered structures [34], [36], approximates the probe as prolate spheroid. The scattered field is determined by $E_{\text{sca}}=\alpha_{\text{eff}}{\left(1+r_{p}\right)}^{2}E_{\text{inc}}$ , where *α*_eff_ is the effective polarizability of the tip–sample system, *E*_inc_ is the incident field, and *r*_*p*_ is the far-field reflection coefficient that is often reasonably ignored as it varies slowly relative to the spatial length scales of typical measurements. This assumption is especially valid at THz frequencies with a sub-mm spot size. Within the framework of the FDM,

(1) $$\alpha_{\text{eff}}\propto 1+\frac{1}{2}\frac{\beta f_{0}}{1-\beta \left(\omega ,q\right)f_{1}},$$

where *f*_0,1_ represent geometric functions describing the characteristics of the tip (see the Supplementary Information and references therein for details) and *β* is the complex near-field reflection coefficient that depends on frequency, *ω* and the in-plane momentum, *q* of the electric near-field. Importantly, *β* carries information about the material properties of the sample, including the dielectric permittivity, which in its simplest form is written as *β* = (*ϵ* − 1)/(*ϵ* + 1) (see Supplementary Information for details). Evidently then the process of extracting the material properties follows from an inversion of the scattering problem. This is made more complex by the fact that the height of the tip above the sample surface is not fixed, in order to remove background and isolate the scattered near-field the tip oscillates. Demodulation involves a Fourier decomposition into a series of harmonic orders that describe the overall scattering during a full oscillation cycle. We thus use an algorithmic approach, similar to Refs. [28], [33], where a numerical routine aims to minimize the deviation between measured data and a corresponding scattered signal calculated from the dielectric function using the layer-extended FDM model (see Supplementary Information for further details of the inversion algorithm).

## 3 Results and discussion

We first show WL imaging of a selected nanoribbon in Figure 2c (overview: 9 × 9 μm at 36 nm per pixel) and Figure 2d (zoom-in: 2 × 2 μm at 10 nm per pixel). Here, we choose to present the second-order demodulated scattered near-field contrast, $\eta_{2}={\hat{E}}_{2}^{\text{sample}}/{\hat{E}}_{2}^{\text{ref}}$ , which is normalized to an average substrate response (or reference) corresponding to the mean signal within the white solid boxes, respectively. Relative to the substrate, the MoS_2_ nanoribbon and its crystallites show a significantly lower scattered near-field signal. This indicates that the magnitude of the permittivity of MoS_2_ regions is lower than that of *c*-cut sapphire. More notable are clear variations in contrast along the nanoribbon and between the nanoribbon and certain crystallites, some of which show a strikingly depressed contrast. A transition region between the substrate and the nanoribbon accents the edge. Linecuts through this area of the zoomed WL image, approximately perpendicular to the edge, for demodulation orders 2–4 (see Figure S3b) together with the corresponding linecut through the AFM topography (see Figure S3a) indicate the instruments spatial resolution to be approximately 50 nm. The resolution is principally governed by the sharpness of the tip at its apex (typically 30 nm based on SEM imaging of multiple tips), but it is also known that manifest edge effects can influence the image contrast in s-SNOM, including abrupt changes in the surface topography [48] and the dielectric environment of two dissimilar materials (particularly pronounced at the interface of an insulator and a metal, giving rise to an asymmetric transition of the image contrast) [49]. Although we see no clear asymmetry in our transition region across the nanoribbon edge over all inspected orders of the demodulated signal (due to the relatively small change in the dielectric values of the MoS_2_ and the sapphire substrate), we do note a small peak in the relative contrast in the fourth-order scattered signal before it drops moving onto the nanoribbon that can suggest the measurement in this region is weakly influenced by a topographic artifact [48].

**Figure 3:**
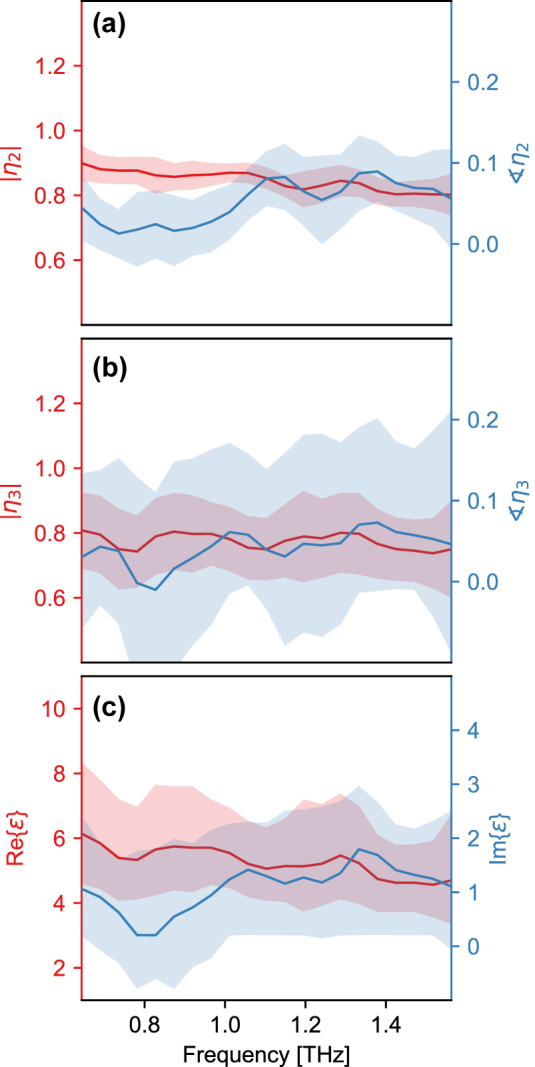
Spectrally resolved THz nanoscopy; (a) second-order contrast and (b) third-order contrast. The solid lines indicate the mean response of 50 adjacent scans at the center of the nanoribbon and the filled area indicates the corresponding standard deviations. (c) Extracted effective complex permittivity spectrum (see Supplementary Information for details of the algorithmic minimization procedure).

Next we perform THz nanoscopy along a specific line, as indicated on Figure 2a. The full scattered THz waveform is captured in the time domain, with a spatial step-size of approximately 10 nm, starting and ending on the substrate and traversing the complete short axis of the nanoribbon. As before when WL imaging, the captured signal is demodulated and normalized to a reference comprising an average response of the substrate to obtain a relative contrast for a given harmonic order (see Supplementary Information for details regarding the normalization procedure). The hyperspectral frequency-position data are spatially averaged over the central region of the nanoribbon where we observe little to no dependence in the contrast as a function of position. The resulting complex Fourier spectrum, and its corresponding standard deviation, for orders two and three is reported in Figure 3a and b, respectively. Both the real and imaginary parts of the contrast exhibit a rather featureless spectrum within the finite bandwidth limits of our probe. The strength of the contrast for the higher demodulation order is marginally greater, but this is accompanied by an increase in the standard deviation due to the corresponding reduced signal-to-noise ratio. The imaginary part is small and appears to be a near-constant value just above zero.

To extract the effective complex permittivity from the spectrally dependent scattering contrast, we utilize a numerical minimization procedure to invert the FDM (see the Supplementary Information for details of the algorithm used). Due to the uniaxial anisotropy of the *c*-cut sapphire substrate, its static effective permittivity was modeled by taking the geometric mean of the in-plane and out-of-plane components, $\varepsilon_{{\text{sub}}_{\|}}=9.46$ and $\varepsilon_{{\text{sub}}_{⊥}}=11.68$ [50]. The extracted permittivity shown in Figure 3c has a featureless spectrum, with only small ripples that we attribute to a residual error in the elimination of background contributions to the near-field signal arising from the instruments’ response function [51] and small-scale amplitude fluctuations or phase drift in the light source. Although an additional normalization step by signal harmonics can provide effective suppression of such spectral artifacts at the cost of reduced material contrasts [52], for the specific geometry and dimensions of our isolated nanoribbon normalization to the reference substrate alone yielded the best results. The flat spectral response is indicative of a low carrier density. Furthermore, the lowest frequency infrared-active phonons in single-layer and bulk MoS_2_ reside at frequencies above 10 THz [53], well beyond the bandwidth of our probe and, therefore, we do not expect to be sensitive to even the tails of these structural resonances. In fact, the real-part mean, $Re\left\{\bar{\varepsilon }\right\}=5.43$ , is in excellent agreement with recent measurements of the static permittivity of MoS_2_ thin films performed using EFM [23], [24]. Sensitivity of the extracted complex effective permittivity to input parameters to the FDM that is core to the inversion algorithm is only weakly dependent on the tip radius and tapping amplitude, but more strongly dependent on the dominant in-plane momentum (as highlighted by Figure S8).

**Figure 4:**
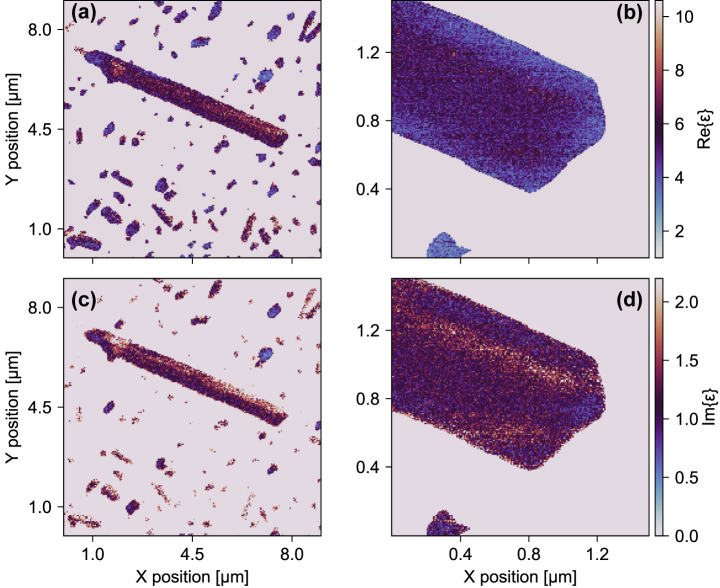
Spatially resolved maps of the real (a, b) and imaginary (c, d) parts of the complex permittivity, extracted from WL imaging of the selected MoS_2_ nanoribbon, overview (a, c) and zoom-in (b, d).

The featureless spectrum, confirmed by our line-scan measurements using detection of the full scattered THz waveform (TDS-mode) to recover amplitude and phase information, allows us to further analyze the WL imaging – where detection recovers a weighted spectrally integrated electric near-field response – in order to investigate nanoscale variations in the effective permittivity. Using the same inversion procedure as introduced above, but this time applied to the WL data (in Figure 2c and d), we show the extracted real (a, b) and imaginary parts (c, d) of the effective complex permittivity in Figure 4a–d for the overview (a, c) and zoom-in (b, d), respectively. Throughout the inversion, we assume a constant value for the phase of the scattered field taken to be the frequency-resolved average from TDS measurements (see the Supplementary Information for details). While this assumption is imperfect, due to the nature of the WL detection being a mix of contrast due to changes in amplitude and phase, we confirm at several spatial locations across the nanoribbon that this approximation is only a small correction to the full response and variations in the measured contrast, leading to a spatial distribution of extracted permittivities, is dominated by contributions from changes in amplitude (see Figure S1). In contrast to recording the full waveform at each spatial pixel, WL detection is relatively fast. Thus, we believe this approach allows us to rapidly determine nanoscale regions within the entire nanoribbon with distinct distributions of the permittivity that would otherwise be obscured using far-field optical probes that at best could perform a spatially averaged response over a small ensemble of nanoribbons or not captured with single-point THz nanoscopy.

**Figure 5:**
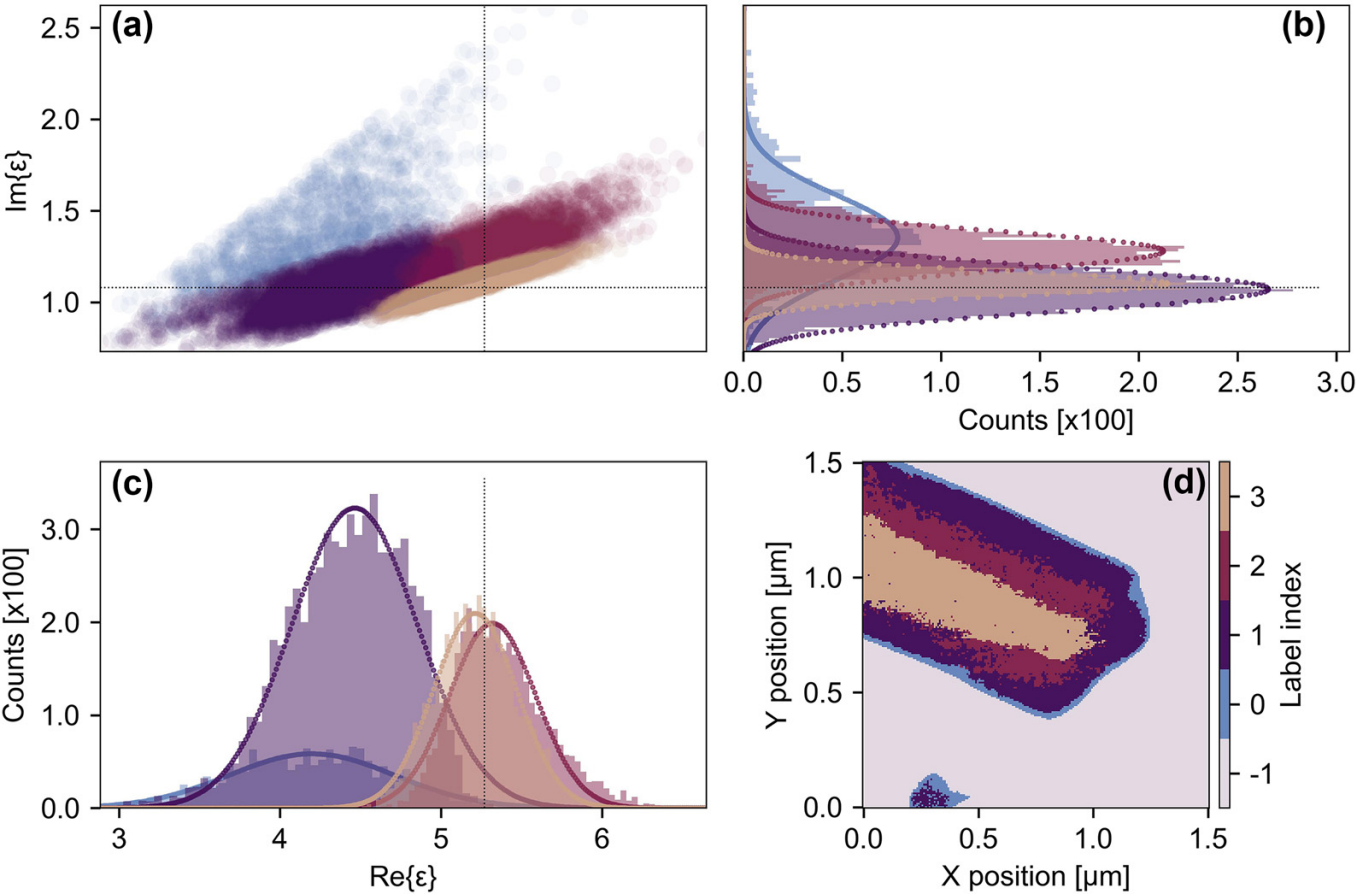
Clustering analysis of the spatially resolved permittivity data. (a) Four distinct clusters in the complex plane. Projections of the four dominant cluster distributions onto the imaginary (b) and real (c) axis. (d) Mapping of the clusters back to real-space to identify variations in the nanoscale permittivity associated with local changes in material properties.

We perform a clustering analysis of the real-space data in order to identify heterogeneities in the effective permittivity at the nanoscale. In Figure 5a, we show a phase-space representation of the output of a Bayesian Gaussian mixture model that identifies four distinct clusters in the complex plane. In Figure 5b and c, these clusters are projected onto the real and imaginary axis and the resulting distributions are fitted with a Gaussian function, respectively. The horizontal and vertical black dotted lines represent the mean values of the real and imaginary parts, as determined from the THz nanoscopy data (Figure 3c). The clusters (indexed 0–3, with −1 being the substrate) are then mapped back onto the real-space image of the nanoribbon (Figure 5d). A narrow band is evident around the edges of the nanoribbon that corresponds to a broadly distributed Gaussian with a center-of-mass significantly shifted from the spectrally averaged mean determined by THz-SNOM (TDS-mode). The spatial distribution of this cluster, localized to the edges of the nanoribbon, agrees well with regions where we identify a high degree of local surface curvature (inferred from the second-derivative of the AFM topography, see Figure S6). The curvature of TMD films determined in this way has previously been connected with areas of increased local strain [40]. Tensile and compressive strain can influence the dielectric properties of materials. A compressive strain less than 10 % in monolayer MoS_2_ has been predicted by calculations to result in a lowered static permittivity [54]; typically, the reduction is on the order of several percent, which cannot fully account for the shift away from the mean observed for this edge cluster. We, therefore, suggest that other influences, including artifacts of the measurement from the sample topography and not from the material properties, convolute the data in this region. The second cluster (with index 1) is larger but shows a similarly shifted distribution in the real-part of the permittivity, well separated from the two final clusters (index 2, 3) that comprise the spatial core of the nanoribbon and show the tightest distribution around the mean based on the nanoscopy data. Importantly, these core clusters of the nanoribbon are distinguishable by their permittivity distributions in the complex plane. We propose that this could arise due to subtle differences in their nanoscale material properties, including variations in local carrier concentration or the spatially integrated density of point defects.

Finally, we note that although the dependence of the extracted effective complex permittivity is essentially independent of the MoS_2_ nanoribbon thickness (see Figure S7), a transition from a lower to a higher real-part permittivity appears to occur around 8–9 nm (corresponding to approximately 13 atomic layers). Recent studies have shown similar tendencies in the increase of the static permittivity with increasing thickness of MoS_2_ [23]. An increased surface-to-volume ratio and reduced dimensionality of materials can be associated with an reduction in both the dielectric permittivity and the scattering time of mobile charge carriers in semiconductors and metals, due to the breaking of polarizable bonds and enhanced scattering – surface effects modifying the optical and electrical properties of materials [55]. This could indicate an evolution of the optical properties from single- and few-layer characteristics to a bulk-like behavior. However, we again note that the thinner regions are proximal to the edges of the nanoribbon and, therefore, may be more strongly dominated by edge effects, as discussed above and only weakly dependent on dimensionality.

## 4 Conclusions

We have quantitatively investigated the nanoscale optical properties of MoS_2_ nanoribbons using THz-SNOM. Initial WL imaging of an isolated nanoribbon, together with satellite crystallites, revealed a clear spatially resolved contrast in the scattered electric near-fields. THz nanoscopy along a cross section of the nanoribbon allowed us to extract the effective complex permittivity that showed a featureless spectrum within our probe bandwidth, well below the lowest frequency resonances of structural phonon modes in the material, indicating a low carrier concentration consistent with the approximately frequency independent behavior of the complex permittivity of a Drude conductor with a low scattering time. By exploiting the phase information from the line scan measurements, where full scattered THz waveforms are detected in the time domain, we are able to extract spatially resolved maps of the complex permittivity of the nanoribbon from WL imaging data, with only a small error due to the rapid detection method being sensitive only to changes in the peak of the scattered THz waveform. The permittivity images reveal significant nanoscale variations in the optical properties of the nanoribbon (and its crystallites). A clustering analysis allows us to resolve four dominant distributions that are separable in the complex plane and map them back into real-space. This allows us to propose mechanisms, such as local strain gradients proximal to the nanoribbon edges or distributions in the spatially averaged density of point defects that could be driving changes to the permittivity over such length scales, and importantly move toward being able to unambiguously distinguish heterogeneities in nanoscale material properties from measurement artifacts, such as topographic effects, known to challenge interpretation of scanning probe techniques, including THz-SNOM. With very few methodologies available for either the direct quantification or indirect evaluation of the complex optical and electrical properties of materials at the nanoscale and the importance of understanding dielectric behavior, including disorder, for the development of opto- and nanoelectronic devices based on layered materials, we believe this approach will be useful for studying many other semiconducting nanomaterial systems in the future.

## Supplementary Material

Supplementary Material Details
