# Supplementary material for: Dielectric permittivity extraction of MoS2 nanoribbons using THz nanoscopy
Source: Nanophotonics. 2025 Apr 25;14(10):1673–82. doi: 10.1515/nanoph-2025-0060 (PMC12116280; doi:10.1515/nanoph-2025-0060)
Supplement: Supplementary file 1 — Supplementary Material Details [file j_nanoph-2025-0060_suppl_001.pdf]

Supplementary Information

Henrik B. Lassen, William V. Carstensen, Denys I. Miakota, Ganesh Ghimire, Stela Canulescu, Peter U. Jepsen, and Edmund J. R. Kelleher*

Dielectric permittivity extraction of MoS_2 nanoribbons using THz nanoscopy

S1 Supporting Information

Supporting information on FDM parameters used for permittivity retrieval, description of minimization routine, and additional analysis of height dependence, strain, harmonic order comparison, and clustering.

S1.1 FDM model and parameters

Simulating the system's response through the FDM requires assumptions about the physical parameters used in the model, such as the tip radius R_t, the effective length of the extended dipole L, the experimentally determined fill factor g, and the in-plane momentum q. This section will discuss the parameter selection used for modelling the THz-SNOM response and ultimately extract the physical sample parameters.

The FDM predicts the scattering contrast, η_i, by comparing the i-th demodulated scattered electric fields from the sample and substrate, respectively. The contrast is directly proportional to the effective polarizability of the tip, such that:

$$\eta_i = \frac{E_i^{sample}}{E_i^{ref}} \propto \frac{\alpha_i^{sample}}{\alpha_i^{ref}}. \tag{S1}$$

The effective polarizability, α, is dependent on the geometry of the system ($f_{0,1}$) and the properties of the sample (β) as shown in the main text Eq. 1. The geometry parameters $f_{0,1}$ are dependent on the tip size, shape, and position above the sample. The height dependence makes the geometry factor time-dependent. The near-field reflection coefficient, β, is derived using the transfer matrix method of Zhan *et al.* [1]. The tip apex radius is estimated from scanning electron microscopy (SEM) images of the standard tips used (Rocky Mountain Nanotechnology 25PtIr200B-40H), with an average of approximately 30 nm. The manufacturer pre-defines the shank length with a total length of 80 μm. The in-plane momentum is determined as the maximum of the weighted distribution of the in-plane momentum distribution given by [2, 3]

$$W(A, R_t, q) = qe^{-2qR_t}e^{q(H+R)}, \tag{S2}$$

using a tip radius of 30 nm, yields a maximum of the weighted distribution at $q = 4.99 \cdot 10^7 \mathrm{m}^{-1}$.

Mooshammer *et al.* showed through spatial representation of the demodulation orders that the effective width of the probe volume decreases with higher orders [4]. The effective in-plane momentum

Henrik B. Lassen, William V. Carstensen, Denys I. Miakota, Ganesh Ghimire, Stela Canulescu, Peter U. Jepsen, Edmund J. R. Kelleher, Department of Electrical and Photonics Engineering, Technical University of Denmark, DK-2800 Kongens Lyngby, Denmark
Henrik B. Lassen, current address: Department of Physics, Kyoto University, Kitashirakawa-Oiwake, Sakyo, Kyoto, Japan

is corrected for by their findings by scaling with the relative change in probing width between the orders. Finally, the permittivity of the substrate, which is selected based on the THz-TDS results of the refractive index of sapphire [5] as discussed in the main text, together with all other model parameters, is summarized in Table S1.

Description	Parameter	Value
**Tip Radius**	R_t	30 nm
**Dipole half-length**	L	40 μm
**In-plane momentum**	q	$2.47 \cdot 10^5$ cm^{-1}
**Tapping amplitude**	A	170 nm
**Substrate permittivity**	$\epsilon_\perp$	11.68
**Substrate permittivity**	$\epsilon_\parallel$	9.46
**Angle of incidence**	θ	30°

Tab. S1: Parameters used in the FDM model for permittivity extraction.

S1.2 Permittivity extraction method

Inversion of THz-SNOM data to permittivity values was performed numerically using a minimization algorithm. The goal of the algorithm was to find the permittivity parameters (ϵ_r, ϵ_i) that resulted in the best overlap between simulation and measurement. The algorithm uses the differential evolution method from SciPy. The error function used for minimization is an expanded version of the one applied by Ritchie *et al.* [6] in combination with the iterative method of Mooshammer *et al.* [7]. The iterative part was dynamically varying permittivity parameters to find the lowest value of the error function and feed it back into the system. Our extension of the Ritchie *et al.* error function consists of two additional terms that allow the algorithm to minimize multiple orders simultaneously. The original two error terms are given as

$$E_{abs,i} = \frac{S_{exp,i} - S_{FDM,i}}{0.5(S_{exp,i} + S_{FDM,i})}, \quad E_{phase,i} = \frac{\phi_{exp,i} - \phi_{FDM,i}}{0.5(\phi_{exp,i} + \phi_{FDM,i})}, \tag{S3}$$

where S_i represents the absolute value and ϕ_i represents the angle of the scattering contrast of the i-th order. When applying the algorithm to multiple orders using only the terms in Eq. S3, it will not minimize for all orders simultaneously, but rather the absolute value of a single order and the phase of a single order. To mitigate this behavior, a penalty is introduced for optimizing better for some orders than others. The penalty is the standard deviation of the errors in the absolute and phase values, respectively

$$E_{penalty} = STD(E_{abs}) + STD(E_{phase}) = \sigma_{abs} + \sigma_{phase}. \tag{S4}$$

The total error function can then be described as

$$E_{tot} = \sum (E_{abs,i} + E_{phase,i}) + \sigma_{abs} + \sigma_{phase}. \tag{S5}$$

This method is applied to each frequency individually and to each pixel individually in the WL images.

When inverting the WL imaging, each pixel value not only represents the absolute value of the frequency average, but is a mix of the phase and amplitude response. This is due to the WL measurement using a fixed time delay for all spatial positions in the scanned area. A phase shift of the pulse moves its peak away from the reference time delay, effectively decreasing the amplitude. As discussed in the main text, the frequency-dependent phase shift was assumed constant, based on inspection of the THz nanoscopy data. By taking the full THz waveform measured on the sample and applying the inverse phase shift, we can estimate the error in WL amplitude caused by a variable phase contribution.

Figure S1 shows the waveforms of the second-order demodulated signal measured on the sample (red) and on the substrate (green) which is used in the main analysis. The average phase shift is applied inversely on the sample measurement recovering the blue signal, which has better temporal overlap with the substrate at the peak. The amplitude difference at the peak between the red and the blue curves is approximately 0.5%.

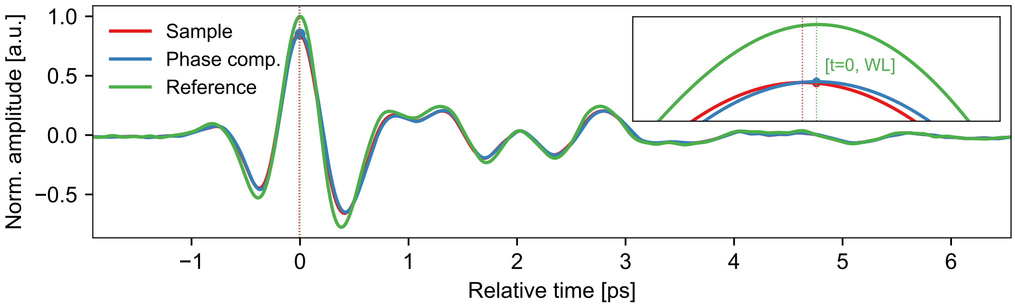

Fig. S1: Second-order demodulated waveforms recorded on the substrate (green) and the sample (red), shown with a phase-corrected sample measurement (blue). The waveform is normalized to the peak of the reference waveform. The inset shows a magnified view of the peak of the waveforms with the vertical lines indicating the peak positions.

S1.3 Permittivity comparison from different harmonic orders

When extracting the permittivity through the minimization routine described above, each harmonic should ideally return the same value, assuming there is no noise. In reality, the signal is noisy, resulting in each order, when used separately, returning slightly different values.

The minimization performed on the orders separately for a cross-section across the zoom-in scan of the nanoribbon is shown in Fig. S2. The central area of the nanoribbon shows a noisy plateau in permittivity, although the topography varies by several nanometers. This suggests that the material parameters are independent of height and that the method of extraction is robust. Using multiple orders will result in the same permittivity with less noise.

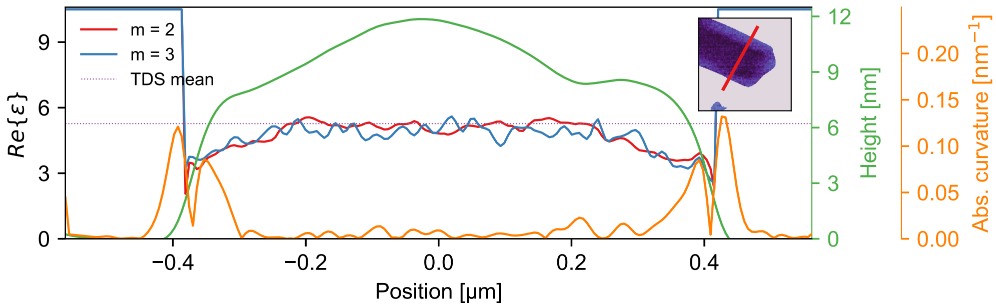

Fig. S2: Line profile taken from the zoom-in permittivity map for the $i = 2$, and $i = 3$ harmonics. The position of the line profile is indicated by the red line on the inset. It should be noted, the extracted profile is not at the same position on the nanoribbon as where the THz nanoscopy was performed, as is discussed in the main text. The height from the substrate (or sample thickness) and curvature are shown in green and orange.

In Fig. S2, a band of approximately 100 nm around the nanoribbon can be seen with lower permittivity. The orange curve shows the curvature of the nanoribbon, which can indicate a region of increased strain in the material [8], which in turn can affect the local permittivity of the material [9]. The curvature falls off faster than the permittivity recovers when moving toward the center of the nanoribbon. This implies that the lowered permittivity cannot be fully explained by the impact of strain causing a change in the permittivity. Furthermore, previous reports have suggested nanoribbons are less prone to a build-up of residual strain, due to different strain-releasing processes during growth [10].

The FDM assumes an infinite and uniform layered stack below the tip. Proximal to edges several artefacts can occur, such as edge darkening, where the effective signal is reduced due to a rapid change in surface topography [11]. The lower signal strength would then result in an effectively lower permittivity. However, the change in WL signal over an edge is wider than the step itself. This is illustrated in Fig. S3. Here the topography and WL signal for orders 2-4 are shown with the 10% and 90% positions of the step height. The width of the step is roughly 60 nm, while the WL signal does not stabilise for another few tens of nanometers. Each order is normalized to one in the substrate. To the left of the edge, all values for the third and fourth orders are greater than one, suggesting that all the data immediately to the left of the nanoribbon is impacted by the nearby edge.

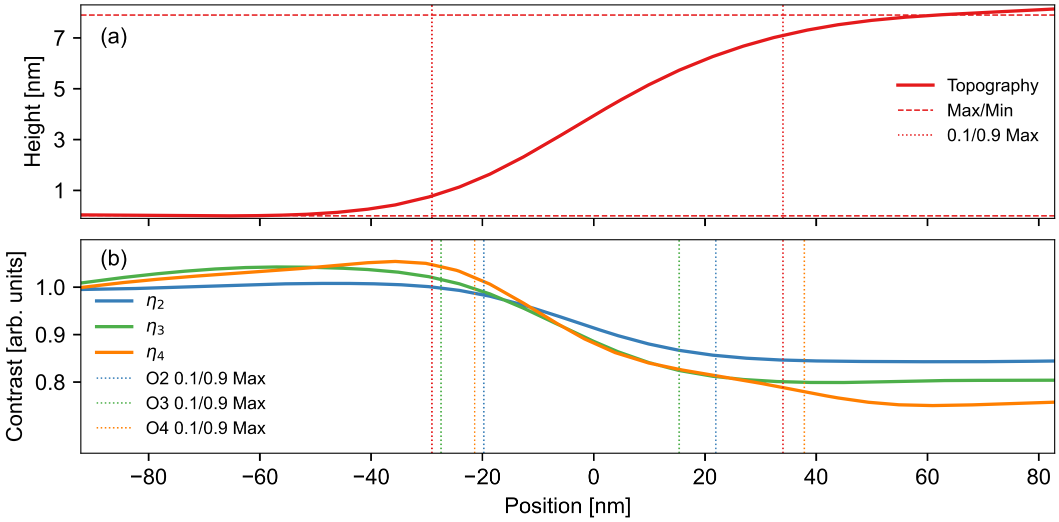

Fig. S3: (a) AFM topography over an edge shown with the 10% and 90% positions of the lower and upper plateaus. (b) THz-SNOM WL contrast for orders 2-4 with their respective 10/90% lines.

S1.4 Clustering

Using a combination of WL data from the harmonic orders and the calculated permittivity values, it is possible to perform clustering to identify areas with similar properties. The clustering algorithm used in this work is a Bayesian Gaussian mixture model. This model determines multi-dimensional Gaussian distributions that fit the data and assigns the data to the cluster with the highest probability based on the discovered functions. Based on calculations of the Akaike information criterion using different numbers of clusters, the optimal number of clusters was determined to be four. This number of clusters has a low prediction error while avoiding overfitting the data.

The results of the algorithm on the zoom-in area of the nanoribbon are shown in the main text. Here, the results for the nanoribbon overview are presented (see Fig. S4). This map captures the

satellite MoS_2 crystallites. The close-up scan showed distributions indicating areas where topography and material properties are convoluted, as well as areas with consistent material properties. The overview scan reveals many areas with potentially varying properties.

The clustering for the overview cannot separate the topographically entangled points from the material properties due to the large proportion of these points, lower resolution, and more total edge points. In this case, the clusters cannot be interpreted as areas with constant properties and Gaussian noise but rather as areas with similar results from the entanglement of material properties and topographic effects. However, certain crystallites appear to belong to one cluster (index 0), while others belong to another cluster (index 1). The reason for this is worthly of further investigation, but could indicate a difference in growth conditions, residual local strain, or topography-induced artefacts. Ultimately the signal quality and spatial resolution of the scan limits the spatial separation of the clusters.

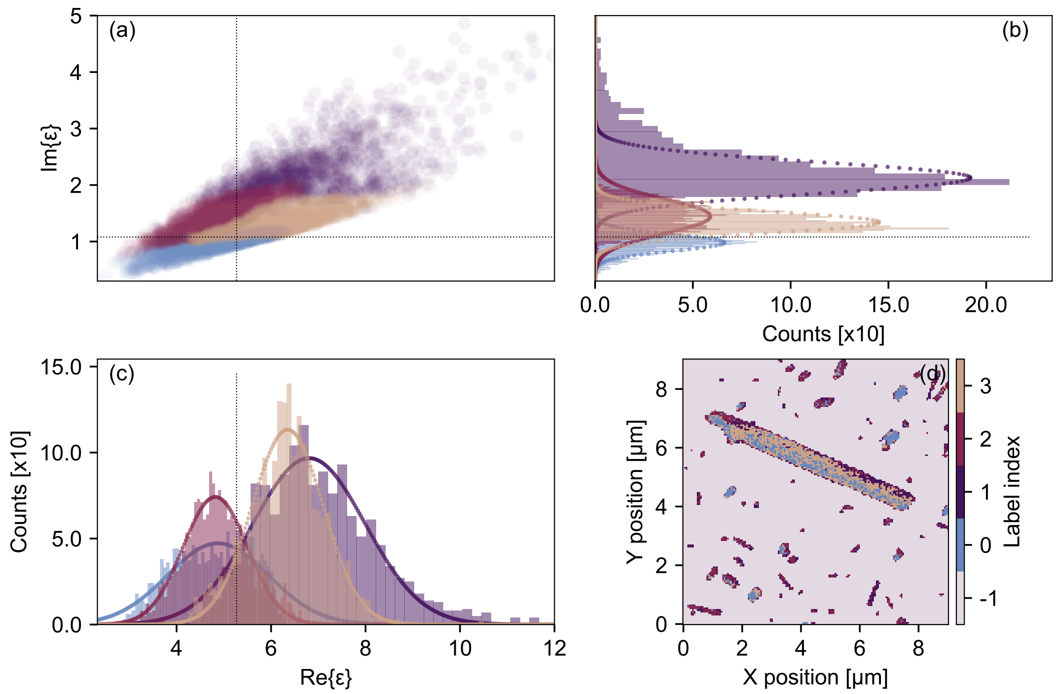

Fig. S4: Clustering analysis of the spatially resolved permittivity data in the WL overview. (a) Four distinct clusters in the complex plane. Projections of the four dominant cluster distributions onto the imaginary (b) and real (c) axis. (d) Mapping of the clusters back to real-space to identify variations in the nanoscale permittivity associated with local changes in material properties.

By making cut-outs from within the nanoribbon core it is possible to analyse nanoscale changes in the permittivity without considering edge artefacts or the influence of strain (due to a relatively constant profile of the curvature throughout the selected regions). The core of the nanoribbon is segmented into three sections as shown in Fig. S5. The corresponding distribution of the real and imaginary parts of the extracted permittivity from each section is plotted and fitted with a single Gaussian function. Each region shows a clearly separated distribution suggesting indeed we capture variations in the effective complex permittivity on sub-micrometer length-scales that could arise due to local changes in carrier or defect concentration. The vertical lines indicate the mean and standard deviation taken from the spectrally inverted data based on a full-field analysis of the scattered THz waveform.

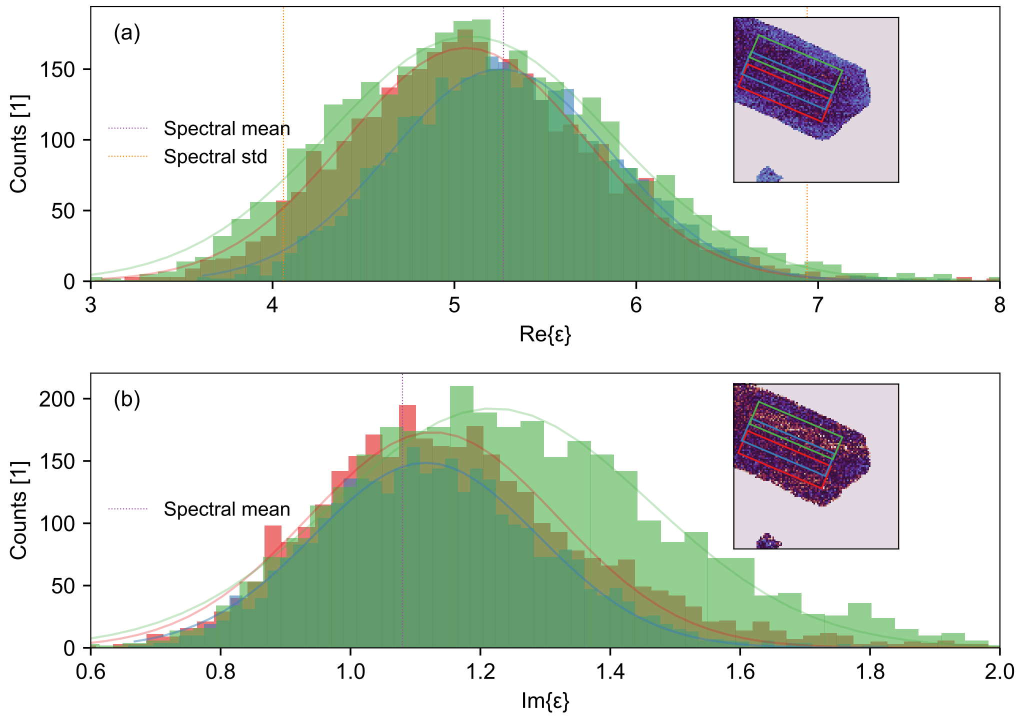

Fig. S5: Real (a) and imaginary (b) permittivity distributions extracted from three overlapping areas (colour-coded to the corresponding distribution) from within the core of the nanoribbon, based on inversion of the WL image of the zoom-in (as indicated in the insets, respectively).

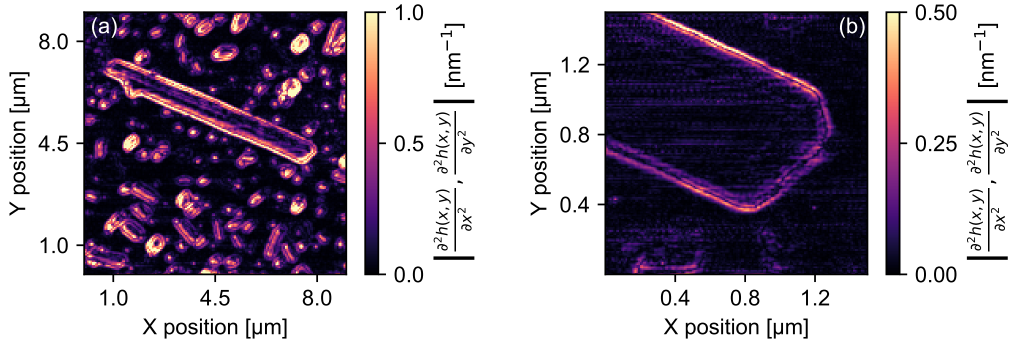

Fig. S6: Spatial maps of the absolute value of the second derivative of the topography for the overview (a) and the zoom-in (b).

S1.5 Curvature and strain

According to Rahaman *et al.*, the curvature of the topographic map can be related to the magnitude of the local strain in a material [8]. By applying the Laplacian to the topographic maps (both the overview and zoom-in) the curvature is extracted and shown in Fig. S6. The curvature is largest near the edges and almost constant and zero across the core of the nanoribbon, this is in agreement with predictions of strain release in nanoribbons during the growth process. The curvature varies between the satellite crystallites suggesting possible differences in local strain induced during growth.

Yue et al. have shown that strain in MoS_2 changes its permittivity depending on the magnitude and direction of the strain [9]. This means the permittivity of the MoS_2 nanoribbon might change near the edges. However, due to the impact of topographic cross-talk affecting the scattered near-

field signal, further clarifying investigations are needed to separate the relative contributions of these various contributions to the observed spatial variations in permittivity.

S1.6 Thickness dependence

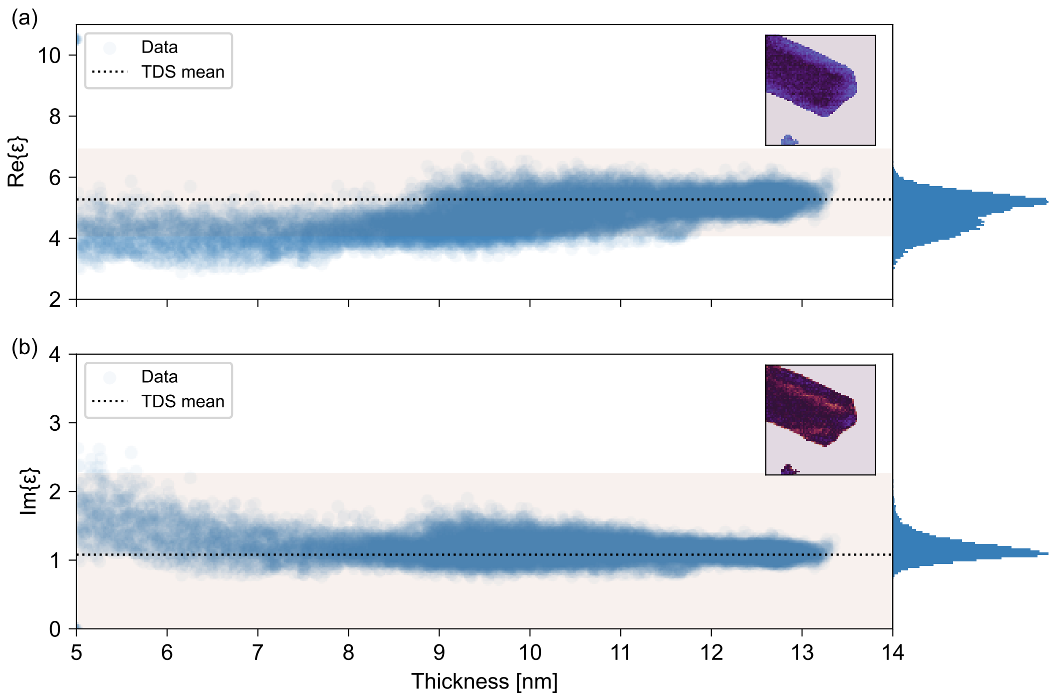

Fig. S7: Thickness dependence of the real (a) and imaginary (b) parts of the permittivity inverted from the zoom-in WL imaging of the nanoribbon. Projections to the right show the corresponding histogram distributions. The horizontal dotted black line indicates the mean value from THz nanoscopy measurements and the corresponding standard deviation (transparent band).

We show how the real and imaginary parts of the extracted permittivity dependent insensitively on the sample thickness (see Fig. S7a and b). A small, but noticeable shoulder around 9 nm in the real-part could indicate a transition to bulk-like behaviour; however, the real-space image suggests the regions with reduced thickness correlated with a reduced real-part permittivity comprise a band proximal to the nanoribbon edge. This once again indicates that cross-talk due to topographic artefacts as the probe transitions between the nanoribbon and substrate make it difficult to draw a strong conclusion without further clarifying investigations.

S1.7 Sensitivity of extracted permittivity to variations in key model parameters

We explore the sensitivity of the inversion algorithm to variations in the FDM input parameters around the optimal values used throughout this study. Figure S8 (a-c) summarizes the impact of three key parameters, including tip radius at a constant in-plane momentum, tapping amplitude, and in-plane momentum for a constant tip radius. While the extracted permittivity is only weakly dependent on the tip radius and tapping ampliude, it is rather more strongly dependent on changes to the dominant in-plane momentum.

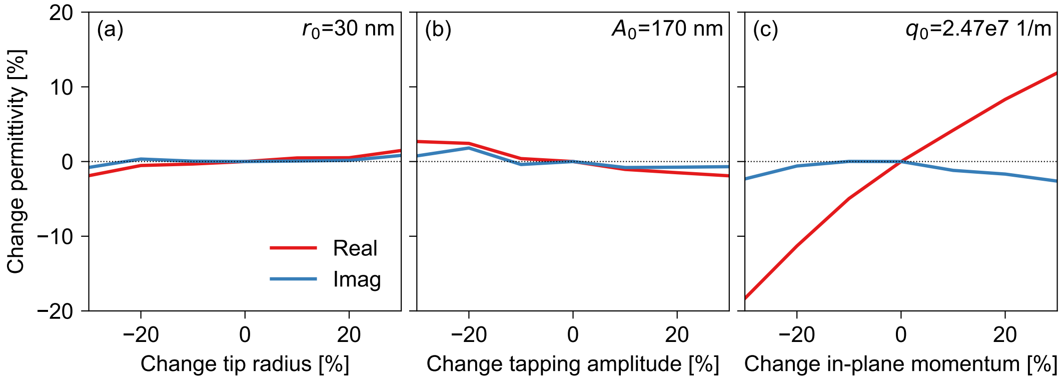

Fig. S8: Percentage change in the complex permittivity based on variation in (a) tip radius at a constant in-plane momentum, (b) tapping amplitude, (c) in-plane momentum for a constant tip radius, as calculated using the FDM.

S1.8 Raw spectral data

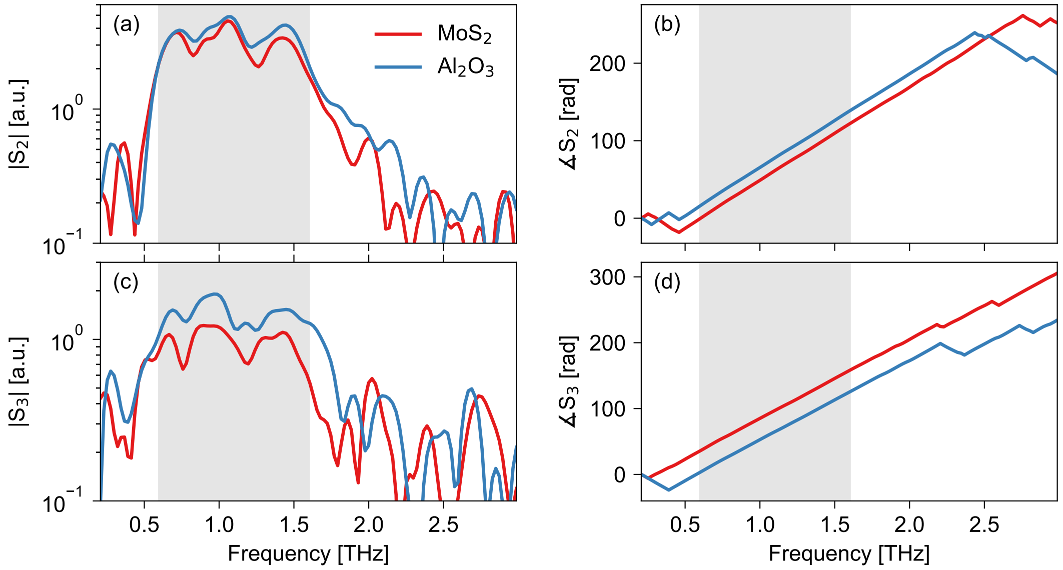

Fig. S9: Raw spectral data. The spectral magnitude of the sample and substrate response (a,c), and the corresponding spectral phases (b,d) for the second (a,b) and third order (c,d) demodulated signals, respectively. The highlighted grey region shows the frequency range over which quantitative analysis is performed. Observed modulations in both sample and reference spectra are attributed to tip-induced artefacts and have minimal impact on subsequent analysis after normalization.

The Rocky Mountain tips used in this study are known to have a strong antenna effect [12], which can introduce frequency dependent artefacts. To identify these, in Fig. S9 we present the raw spectra and corresponding spectral phases of the second and third order demodulated signals from within the core of the nanoribbon (averaged over 50 contiguous scans, we call the sample) and from a region on the substrate (named the reference). Clear modulations in the spectra (see Fig. S9 (a,c)) are present in both the sample and reference, which after normalization have minimal impact on the integrity of further quantitative analysis.

References

[1] T. Zhan, X. Shi, Y. Dai, X. Liu, and J. Zi, "Transfer matrix method for optics in graphene layers," *J. Phys.: Condens. Matter*, vol. 25, p. 215301, 2013

[2] B. Hauer, A. P. Engelhardt, and T. Taubner, "Quasi-analytical model for scattering infrared near-field microscopy on layered systems," *Opt Express*, vol. 20, pp. 13173–13188, 2012.

[3] B. Wang and C. H. Woo, "Atomic force microscopy-induced electric field in ferroelectric thin films," *J. Appl. Phys.*, vol. 94, no. 6, pp. 4053–4059, 2003.

[4] F. Mooshammer, M. A. Huber, F. Sandner, M. Plankl, M. Zizlsperger, and R. Huber, "Quantifying nanoscale electromagnetic fields in near-field microscopy by fourier demodulation analysis," *ACS Photonics*, vol. 7, no. 2, pp. 344–351, 2020.

[5] D. Grischkowsky, S. Keiding, M. V. Exter, and C. Fattinger, "Far-infrared time-domain spectroscopy with terahertz beams of dielectrics and semiconductors," *J. Opt. Soc. Am. B*, vol. 7, pp. 2006–2015, 1990.

[6] E. T. Ritchie, C. B. Casper, T. A. Lee, and J. M. Atkin, "Quantitative local conductivity imaging of semiconductors using near-field optical microscopy," *J. Phys. Chem. C*, vol. 126, pp. 4515–4521, 2022.

[7] F. Mooshammer, *et al.*, "Nanoscale near-field tomography of surface states on (Bi0.5Sb0.5)2Te3," *Nano Lett.*, vol. 18, no. 12, pp. 7515–7523, 2018.

[8] M. Rahaman, R. D. Rodriguez, G. Plechinger, S. Moras, C. Schüller, T. Korn, and D. R. Zahn, "Highly localized strain in a MoS2/Au heterostructure revealed by tip-enhanced raman spectroscopy," *Nano Lett.*, vol. 17, no. 10, pp. 6027–6033, 2017.

[9] Q. Yue, *et al.*, "Mechanical and electronic properties of monolayer MoS2 under elastic strain," *Phys. Lett. A*, vol. 376, no. 12, pp. 1166–1170, 2012.

[10] D. I. Miakota, G. Ghimire, R. Kumar Ulaganathan, M. E. Rodriguez, and S. Canulescu, "A novel two-step route to unidirectional growth of multilayer MoS2 nanoribbons," *Appl. Surf. Sci.*, vol. 619, p. 156748, 2023.

[11] T. Taubner, R. Hillenbrand, and F. Keilmann, "Performance of visible and mid-infrared scattering-type near-field optical microscopes," *J. Microsc.*, vol. 210, no. 3, pp. 311–314, 2003.

[12] F. Mooshammer, M. Plankl, T. Siday, M. Zizlsperger, F. Sandner, R. Vitalone, R. Jing, M. A. Huber, D. N. Basov, and R. Huber, "Quantitative tereahertz emission nanoscopy with multiresonant near-field probes," *Opt. Lett.*, vol. 46, no. 15, pp. 3572–3575 (2021).
